# Resin-based sealant effectiveness in high-caries risk children: a systematic review

**DOI:** 10.1186/s12903-025-06158-0

**Published:** 2025-05-23

**Authors:** Yodsapat Paemanukornruk, Nicha Luksamijarulkul, Piyada Gaewkhiew

**Affiliations:** Department of Community Dentistry, Faculty of Dentistry, Mahidol University, Bangkok, 10400 Thailand

**Keywords:** Sealant, Children, Caries risk, Caries

## Abstract

**Background:**

There have been limited previous reviews on the use of sealants in high-caries-risk children that include multiple study designs. This systematic review aimed to evaluate the preventive effect of sealants on high-caries-risk children and compare the effectiveness of sealants between high-caries-risk children and lower-caries-risk children.

**Methods:**

An initial protocol was developed following the PRISMA guidelines. A comprehensive literature search was performed across several electronic databases, including PubMed, the Cochrane Library, Embase and Google Scholar. Experimental or observational studies that examined resin-based sealants for preventing caries over at least 12 months in high-caries-risk children, identified by individual risk assessment, were included. Comparison groups were moderate- or low-risk children or no sealant; if unavailable, only intervention data were used. The risk of bias was assessed via the RoB2 tool and the Newcastle‒Ottawa Scale.

**Results:**

A total of 1651 unique records were identified, 20 of which were included in this systematic review. Studies comparing only sealed teeth among different caries risk groups have indicated that high-caries-risk children have a greater risk of developing new carious lesions than low- or moderate-risk children do. In contrast, the effect of sealants was negligible in low-caries-risk children when compared between sealed and nonsealed teeth. Additionally, caries experience was the primary criterion used across all included studies.

**Conclusion:**

Resin-based sealants are still recommended for high-caries-risk children. However, generalizability and an appropriate threshold for risk assessment remain unclear owing to the limited number of studies from low- to middle-income countries and variability in risk assessment methods.

**Systematic review registration:**

PROSPERO Registration number CRD42023473013.

**Clinical trial number:**

Not applicable.

**Supplementary Information:**

The online version contains supplementary material available at 10.1186/s12903-025-06158-0.

## Introduction

Dental caries is the most prevalent disease in the human population [1]. In particular, among children and adolescents, dental caries impacts the daily lives of both affected children and their family members [2, 3]. In the permanent dentition of children and adolescents, the occlusal surface was consistently the most commonly affected surface, regardless of age, sex, race, or ethnicity [4]. Dental sealants, especially resin-based sealants, have been recommended to reduce the incidence of dental caries on the occlusal surfaces of permanent teeth [5]. Although glass ionomer and compomer sealants are also available, they have lower retention rates than resin-based sealants do, making resin-based sealants the primary material used for sealing pits and fissures [6, 7].

Past reviews and guidelines have gathered research data on sealant use in children at high risk for developing caries [8–11]. However, most of these studies focused only on studies that used economic evaluations of sealant programs and were typically conducted in high-income countries due to greater data integrity. As a result, the generalizability of the findings to low- and middle-income countries is limited, as the health coverage system and incidence of caries differ across countries [12, 13]. Only one review included all study designs, but those studies were published before 2000 [11] which may not reflect the current situation where fluoride utilization is more widespread [14]. Moreover, studies in previous reviews have used various risk assessment methods at the tooth, individual, and community levels [15–19]. This variation makes it difficult for practitioners to choose the most suitable risk assessment method before applying sealants.

This systematic review aims to address these gaps by examining studies published after 2000 that used clearly defined individual caries risk assessment methods. The objective of this review was to evaluate the preventive effect of sealants on high-caries-risk children and compare the effectiveness of sealants between high-caries-risk children and lower-caries-risk children. This review seeks to clarify the practical application of high-risk strategies in sealant programs.

## Methods

### Eligibility criteria

Following the Participants-Intervention-Comparison-Outcome-Study design (PICOS) schema, we included studies that met the following criteria: Participants were high-caries-risk children identified through individual caries risk assessments. The intervention was resin-based sealant application. The comparison groups either included resin-based sealants applied to moderate- or low-caries-risk children or had no sealant. In cases where no suitable control group met the criteria, only data from the intervention group were extracted. The outcomes measured the preventive effect of resin-based sealants on caries, specifically caries incidence, survival rate, and cost-effectiveness. The cost-effectiveness outcome was included only if effectiveness was assessed based on caries incidence, with a minimum follow-up period of 12 months. The study design included experimental or observational studies that fit these parameters.

We excluded in vitro studies, studies involving participants with initial tooth defects such as dental fluorosis or enamel hypoplasia, studies using nonspecific sealant types, studies that combined sealants with other interventions like restorations or fluoride treatments, and studies that applied sealants to proximal surfaces.

### Information sources and literature search

We systematically searched three databases, PubMed, EMBASE Ovid, and the Cochrane Central Register of Controlled Trials (CENTRAL), via the search strategy detailed in Appendix A [see Additional file 1]. Additionally, we manually searched Google Scholar and the reference/citation lists of the included trials for any additional relevant studies. Our inclusion criteria were limited to articles published in English or Thai from 2000 through September 2023.

### Selection process

Two authors (YP, PG) independently screened the search results from the abstract to the full text using predefined eligibility criteria. In the case of disagreements, the third reviewer (NL) resolved conflicts through discussion with all reviewers.

### Data collection process and data items

Two authors (YP, PG) independently extracted the data via a structured data extraction form. This form included study characteristics (study design, year of study, country), patient characteristics (age, method of caries risk assessment), intervention details (comparisons, length of follow-up), and outcome measures (caries incidence).

### Study risk of bias assessment

Two authors (YP, PG) independently assessed the risk of bias via the following tools: the revised Cochrane risk-of-bias tool for randomized trials (RoB 2) for randomized clinical trials, the Risk of Bias in Nonrandomized Studies of Interventions (ROBINS-I) for clinical trials, and the Newcastle‒Ottawa Scale (NOS) for cohort studies.

### Synthesis methods

Study designs were categorized following the measurements of the exposures and interventions as well as the main outcomes (caries incidence). However, a meta-analysis is not feasible due to the variation in exposures and main outcomes. Thus, descriptive analysis was reported.

## Result

**Fig. 1:**
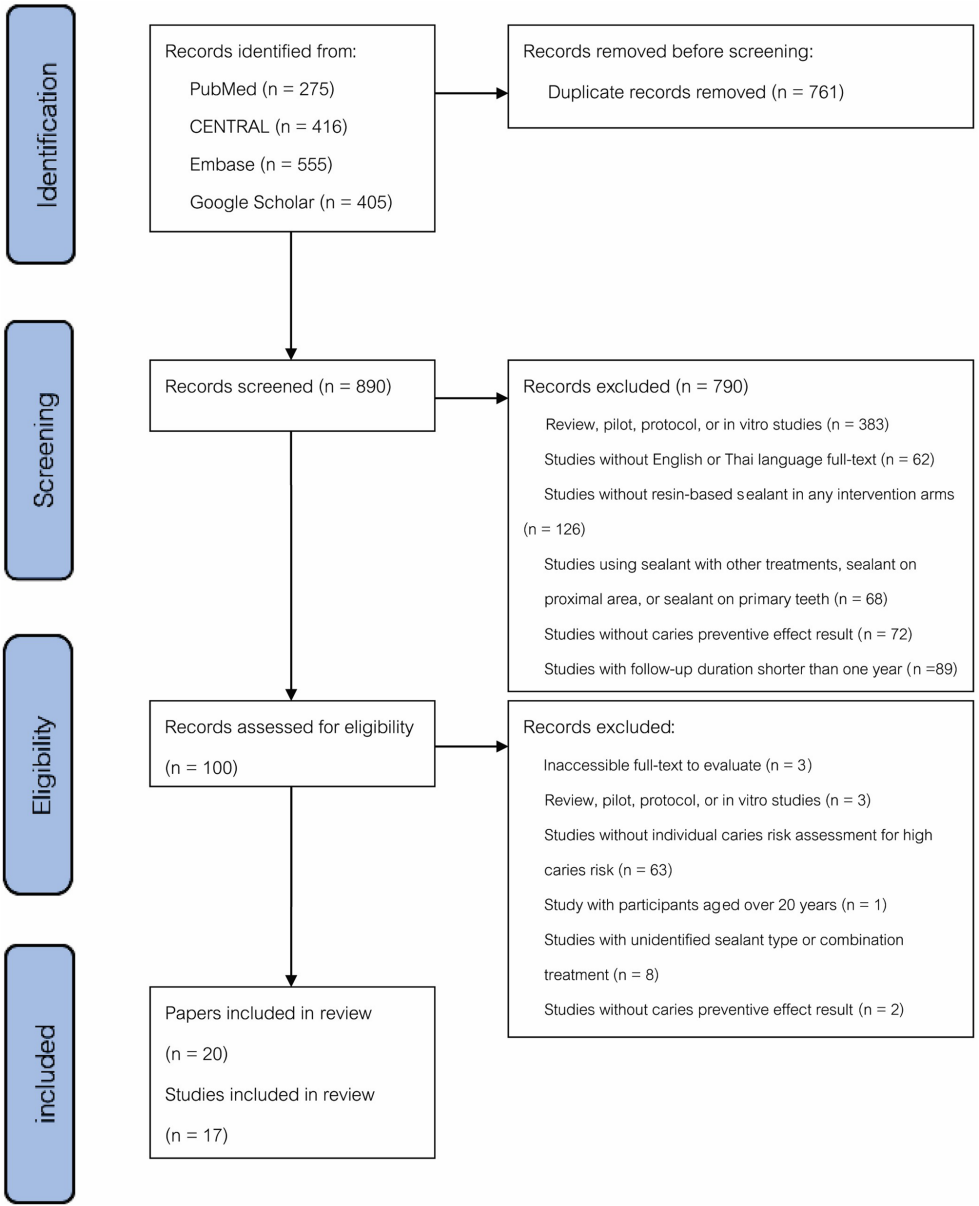
Study flow diagram

### Study selection

A total of 1,651 papers were identified through electronic searches (Fig. 1). After removal of duplicates and initial screening by title or abstract, 102 papers were assessed using the eligibility criteria, and 20 papers were finally included in the systematic review. These 20 papers represent 17 unique studies, as six papers originate from three studies reporting outcomes at different follow-up periods.

### Study characteristics

Five studies conducted statistical analyses across different caries risk groups, as shown in Table 1 [see Additional file 2]. Among these, 60% (3 studies) were split-mouth randomized clinical trials, and 40% (2 studies) were cohort studies, conducted in five different countries. They included 4,575 children (median 276; range 42–3,816) aged 5–9 years. Most studies (*n* = 3; 60%) used decayed, missing, filled teeth (dmft) as a predictor, whereas the remainder (*n* = 2; 40%) used caries experience combined with other factors. The interventions compared included comparisons between different caries risk levels in sealed teeth, sealed versus nonsealed teeth in different risk groups, and various sealing strategies. The follow-up period ranged from 12 to 84 months.

**Table 1:** Studies with statistical analysis across different caries risk groups [22, 39, 40, 49]

Authors, Year of publication	Study design	Study sample	Caries risk grouping criteria	Intervention (sealant brand)	Length of follow-up (months)	Outcome
**Al-Jobair et al.**,** 2017 **[20]	Split-mouth randomized clinical trial	42 children aged 6–9 years in Saudi Arabia (168 permanent first molars: free of caries, restorations, or sealant); 16.7% attrition	Moderate risk (dmft = 1–4), High risk (dmft > 4) [22]	Moderate vs. high caries risk in resin-based sealant (Clinpro™) and GI sealant (GC Fuji Triage)	18	Caries incidence (Oral health survey: basic methods 4th ed.) (Ref: resin sealant in moderate risk) RR in high risk = 24.00 (3.39-169.92) ^a^
**Chen & Liu**,** 2013 **[21]	Split-mouth randomized clinical trial	61 children aged 6–8 years in China (158 permanent first molars: no caries found by visual inspection and probing); 6.6% attrition	Low risk (dmft < 2), High risk (dmft > 5) [39, 40]	Low vs. high caries risk in resin-based sealant (Concise) and GI sealant (GC Fuji VII)	24	Caries incidence (WHO) (Ref: resin sealant in low risk) RR in high risk = 8.89 (1.09–72.20) ^a^
**Muller-Bolla et al.**,** 2016 **[24]; **Muller-Bolla et al.**,** 2013 **[23]	Split-mouth randomized clinical trial	276 children aged 6–7 years in France (914 permanent first molars: ICDAS II codes 0–2 without sealants); attrition 17.4%	Criteria followed Haute Autorite´ de Sante´ method [49]	Sealed teeth (Delton plus) vs. non-sealed teeth	36	Caries incidence (ICDAS II codes 3–6) (Ref: non-sealed teeth) HR in sealed teeth = 0.33 (0.24–0.46) HR in sealed teeth of children with caries at baseline = 0.32 (0.23–0.46) HR in sealed teeth of children without caries at baseline = 0.42 (0.16–1.12)
	Split-mouth randomized clinical trial	276 children aged 6–7 years in France (914 permanent first molars: ICDAS II codes 0–2 without sealants); attrition 8.3%	Criteria followed Haute Autorite´ de Sante´ method [49]	Sealed teeth (Delton plus) vs. non-sealed teeth	12	Caries incidence (ICDAS II code 3–6) (Ref: non-sealed teeth) OR in sealed teeth = 0.55 (0.37–0.81) OR in sealed teeth of children with caries at baseline = 0.25 (0.12–0.50) OR in sealed teeth of children without caries at baseline = 0.32 (0.06–1.63)
**Oulis & Berdouses**,** 2009 **[22]	Cohort	380 children aged 6–8 years in Greece (1,274 permanent first molars: no decalcification or cavitation); attrition 12.5%	Low risk (dmft = 0), Moderate risk (dmft = 1–4), High risk (dmft > 4) (N/A)	Low vs. moderate vs. high caries risk (Delton^®^)	36	Caries incidence (N/A) (Ref: low risk) RR in moderate risk = 1.41 (0.82–2.43) ^a^ RR in high risk = 1.90 (1.34–2.69) ^a^
**Leskinen et al.**,** 2008 **[25]; **Leskinen et al.**,** 2008 **[18]	Retrospective cohort	3816 children aged 6–7 years in Finland; attrition was not reported	Had signs of dental caries and high Streptococcus mutans level in saliva at the age of 2 years. (N/A)	Sealed teeth (Delton plus) vs. non-sealed teeth in high-caries risk sealing strategy and all patient sealing strategy	84	Survival rate at 13 years of age (caries had reached dentin) High-caries risk sealing strategy sealed teeth ≈ 75% ^a^ non-sealed teeth ≈ 80% ^a^ All patient sealing strategy sealed teeth ≈ 75% ^a^ non-sealed teeth ≈ 50% ^a^
	Retrospective cohort	3816 children aged 5–7 years in Finland; attrition was not reported	Had signs of dental caries and high *Streptococcus mutans* level in saliva at the age of 2 years. (N/A)	High-caries risk sealing strategy vs. all patient sealing strategy (Delton)	60	Cost of total treatment per subject (€) High-caries risk sealing strategy = 184.2 All patient sealing strategy = 234.3

dmft: Decayed, Missing, and Filled primary teeth, ICDAS II: International Caries Detection and Assessment System, WHO: World Health Organization, GI: Glass Ionomer, Ref: Reference group, N/A: Reference of the criteria was not mentioned in the paper

^a^**=** calculated from the incidence provided in the result of papers

Table 2 [see Additional file 2] summarizes studies focusing solely on high-caries-risk participants, with 58% (7 studies) being split-mouth randomized clinical trials, 33% (4 studies) being randomized clinical trials, and 8% (1 study) being a clinical trial. These studies were conducted across nine different countries. A total of 2,118 children were included (median 120; range 36–599, one study unreported), aged 5–16 years. All studies used caries experience as an inclusion criterion for high-caries-risk participants, either as a standalone factor (*n* = 5; 42%) or in combination with other factors (*n* = 7; 58%). Interventions compared resin-based with glass-ionomer (GI) sealants (54%), conventional with modified resin sealants (23%), and sealed with nonsealed teeth (15%), while 7% of studies assessed the effects of sealant across different levels of baseline caries progression. The follow-up period ranged from 12 to 60 months.

**Table 2:** Studies that include participants with a high caries risk as part of their inclusion criteria [24, 41, 42, 43, 50]

Authors, Year of publication	Study design	Study sample	Caries risk inclusion criteria (Ref)	Intervention (sealant brand)	Length of follow-up (months)	Outcome
**Gyati et al.**,** 2023 **[34]	Split-mouth randomized clinical trial	Children aged 6–9 years in India (80 permanent first molars: ICDAS II code 0 and without any history of preventive treatment from the last 6 months on the respective teeth); attrition 0%	High caries risk status from AAPD caries risk assessment tools [41]	Hydrophilic sealant (Embrace WetBond) vs. Hydrophobic sealant (Clinpro)	18	Caries progression (ICDAS II) ICDAS II code 0 = 58 (72.5%) ICDAS II code 1 = 18 (22.5%) ICDAS II code 2 = 4 (5%) Total caries incidence if specify at ICDAS II codes 3–6 = 0% ^a^
**Beresescu et al.**,** 2022 **[26]	Clinical trial	119 children aged 6–8 years in Romania (427 permanent first molars: ICDAS II codes 0–3 without sealants or restorations); attrition 4.7%	High caries risk status from CAMBRA system [42]	Baselined ICDAS II (Helioseal F™)	24	Caries incidence (ICDAS II codes 4–6) from baselined ICDAS II codes 0–3 at 24 months = 45 (11.06%) ^a^ at 18 months = 28 (6.88%) ^a^ at 12 months = 17 (4.18%) ^a^
**Kamath et al.**,** 2022 **[29]	Split-mouth randomized clinical trial	62 children aged 6–9 years in India (116 permanent first molars: no discoloration, restorations, cavitations or developmental defects); attrition 6.5%	dft = 3–6 (N/A)	Conventional (Delton FS+) vs. nanofilled (Filtek Z350 flowable nanocomposite)	18	Caries incidence (CCC) Conventional and nanofilled resin sealant at 18 months = 14 (12.07%) ^a^ Conventional and nanofilled resin sealant at 12 months = 11 (9.48%) ^a^
**Tahani et al.**,** 2021 **[30]	Split-mouth randomized clinical trial	124 children aged 7–9 years in Iran (370 permanent first molars: ICDAS II codes 0–2 without dental fluorosis, sealants or restorations); attrition 12.1%	primary molar dmft ≥ 1, not having dental visit at least once a year and not brushing at least once or twice every day [50]	Sealed (Prime-Dent) vs. non-sealed	12	Caries incidence (ICDAS II codes 3–6) from baselined ICDAS II codes 0-2 Sealed teeth = 9 (5.52%) ^a^ Non-sealed teeth = 26 (15.95%) ^a^
**Amaechi et al.**,** 2019 **[31]	Split-mouth randomized clinical trial	120 children aged 7–20 years in the United States (288 permanent molars or premolars: sound, or incipient caries limited to the enamel without sealants or restorations in pits and fissures); attrition 5%	Moderate to high caries risk status from ADA caries risk assessment tools [43]	Selenium-containing (DenteShield™) vs. selenium-free (UltraSeal™ XT Plus)	12	Caries incidence (incipient/ cavitated caries) Selenium-containing and selenium-free sealant = 0 (0%)
**Haricharan et al.**,** 2019 **[27]	Split-mouth randomized clinical trial	90 children aged 7–11 years in India (180 permanent mandibular first molars: no cavitated carious dentinal lesions or developmental anomalies); attrition 0%	DMFT ≥ 2, poor oral hygiene status, history of irregular dental visits and a high frequency of exposure to sugars (N/A)	Moisture-tolerant resin sealant (Embrace WetBond) vs. ART sealant (Ketac Molar Easy Mix)	12	Caries incidence (Oral health survey: basic methods 4th ed.) (Simonsen score 2 or 4) Resin sealant = 28 (31.11%)^a^
**Muller-Bolla et al.**,** 2018 **[35]	Split-mouth randomized clinical trial	400 children aged 5–15 years in France (1,326 permanent molars: ICDAS II codes 0–2 without sealants or restorations); attrition 27.5%	At least one ICDAS II codes 3–6 lesion at baseline [24]	Sealed (Delton plus^®^) vs. non-sealed	24	Caries incidence (ICDAS II codes 3–6) (Ref: non-sealed teeth) HR in sealed teeth = 0.17 (0.15–0.20)
**Hilgert et al.**,** 2017 **[28]	Randomized clinical trial	123 children aged 6–7 years in Brazil (238 occlusal surfaces of permanent first molars: ICDAS II code 1 with medium or deep fissures, ICDAS II code 2, or ICDAS II code 3); attrition 30.1%	≥ 2 cavitated dentine caries in primary molars assessed according to the ICDAS II index (N/A)	Resin-based sealant (Fluoroshield) vs. ART sealant (Ketac Molar Easymix)	36	Cumulative survival (ICDAS II codes 4–6) Resin sealant at 36 months = 91.4% Resin sealant at 24 months = 95.4% Resin sealant at 12 months = 98.1%
**Haznedaroğlu et al.**,** 2016 **[32]	Randomized clinical trial	40 children aged 7–10 years in Turkey (160 permanent first molars: DIAGNOdent-pen score < 13); attrition 40%	a mean dft index ≥ 2 (N/A)	Resin-based sealant (Ultraseal XT^®^) vs. GI sealant (Fuji Triage^®^)	48	Caries incidence (DIAGNOdent-pen score > 20) Resin sealant at 48 months = 12 (21.4%) Resin sealant at 36 months = 0 (0%) Resin sealant at 24 months = 0 (0%) Resin sealant at 12 months = 0 (0%)
**Zhang et al.**,** 2014 **[38]; **Chen et al.**,** 2012 **[33]	Randomized clinical trial	405 children aged 7–9 years in China (1,304 permanent first molars: no dentin caries lesion in pits and fissures); attrition 9.9%	a mean dmfs ≥ 2 (N/A)	Resin-based (Clinpro^®^) vs. GI (Ketac Molar Easymix^®^) vs. GI plus added energy vs. glass carbomer sealant (Glass Carbomer^®^)	48	Cumulative survival (ART caries assessment code2-4) Resin sealant = 96.4%
	Randomized clinical trial	407 children aged 7–9 years in China (1,352 permanent first molars: no dentin caries lesion in pits and fissures); attrition 2.7%	dmft ≥ 2 (N/A)	Resin-based (Clinpro^®^) vs. GI (Ketac Molar Easymix^®^) vs. GI plus added energy vs. glass carbomer sealant (Glass Carbomer^®^)	24	Cumulative survival (ART caries assessment code2-4) Resin sealant = 98.9%
**Barja-Fidalgo et al.**,** 2009 **[37]	Randomized clinical trial	36 children aged 6–8 years in Brazil (92 permanent first molars: sound occlusal surface or noncavitated enamel lesion); attrition 44.4%	primary molar dmft ≥ 2 (N/A)	Resin-based (Delton) vs. GI sealant (Fuji IX)	60	Caries incidence (a cavity that had clearly penetrated the dentin or seen on the bitewing X-ray) Resin sealant = 7 (25%)
**Kervanto-Seppälä et al.**,** 2008 **[36]	Split-mouth randomized clinical trial	599 children aged 12–16 years in Finland (2,356 s permanent molars: no sealants, dentin caries, or restorations); attrition 20%	earlier dentin caries or increased caries activity of a sibling (N/A)	Resin-based (Delton^®^) vs. GI sealant (Fuji III^®^)	36	Caries incidence (dentin caries could be detected) Resin sealant = 7 (1.1%)

dmft: Decayed, Missing, and Filled primary Teeth, dft: Decayed and Filled primary Teeth, dmfs: Decayed, Missing, and Filled Surfaces, DMFT: Decayed, Missing, and Filled permanent Teeth, ICDAS II: International Caries Detection and Assessment System, GI: Glass Ionomer, ART: Atraumatic Restorative Treatment, AAPD: American Academy of Pediatric Dentistry, ADA: American Dental Association, CAMBRA: Caries Management by Risk Assessment, Ref: Reference group, N/A: Reference of the criteria was not mentioned in the paper

^a^**=** calculated from the incidence provided in the result of papers

### Risk of bias in studies

**Fig. 2:**
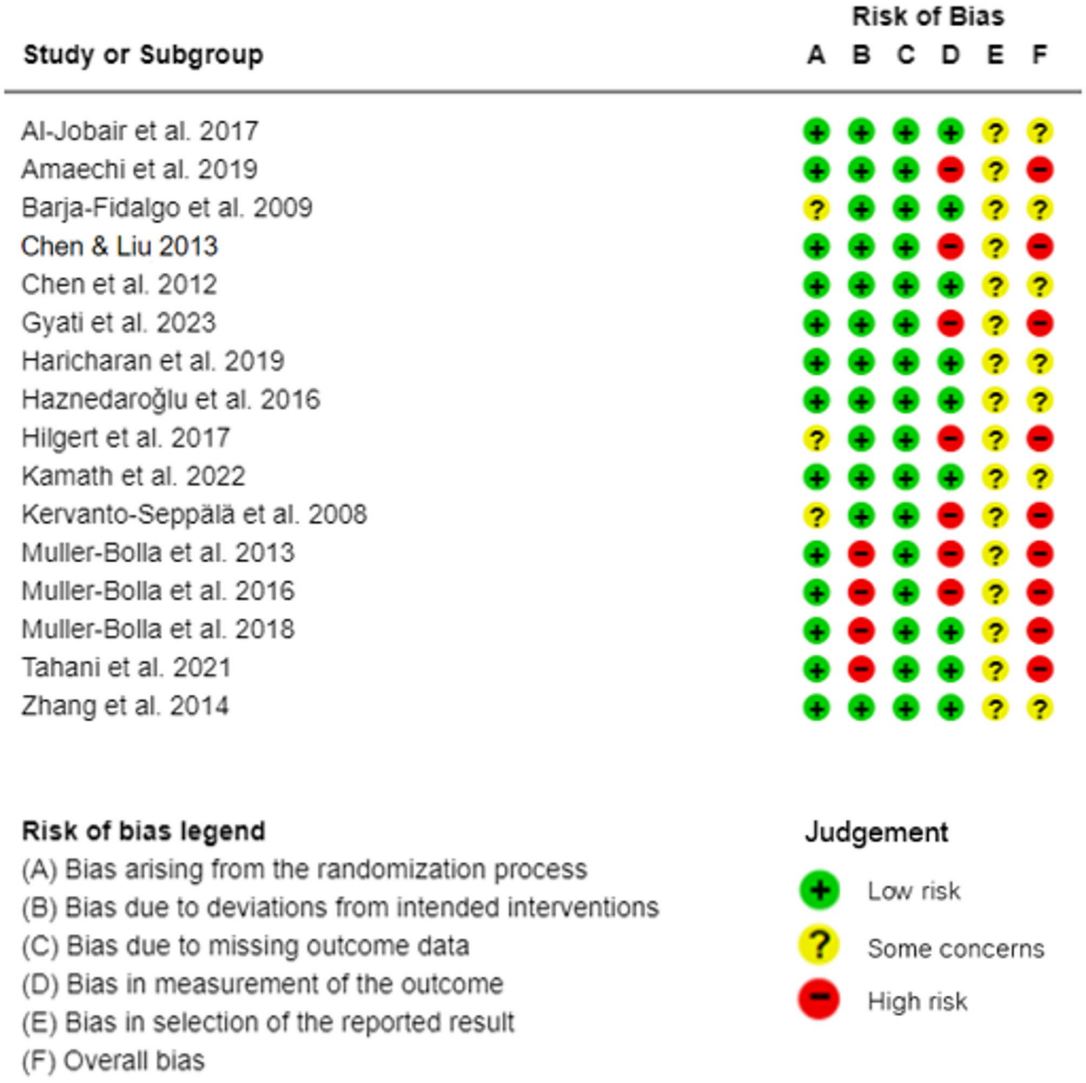
Risk of bias of randomized clinical trials

**Fig. 3:**
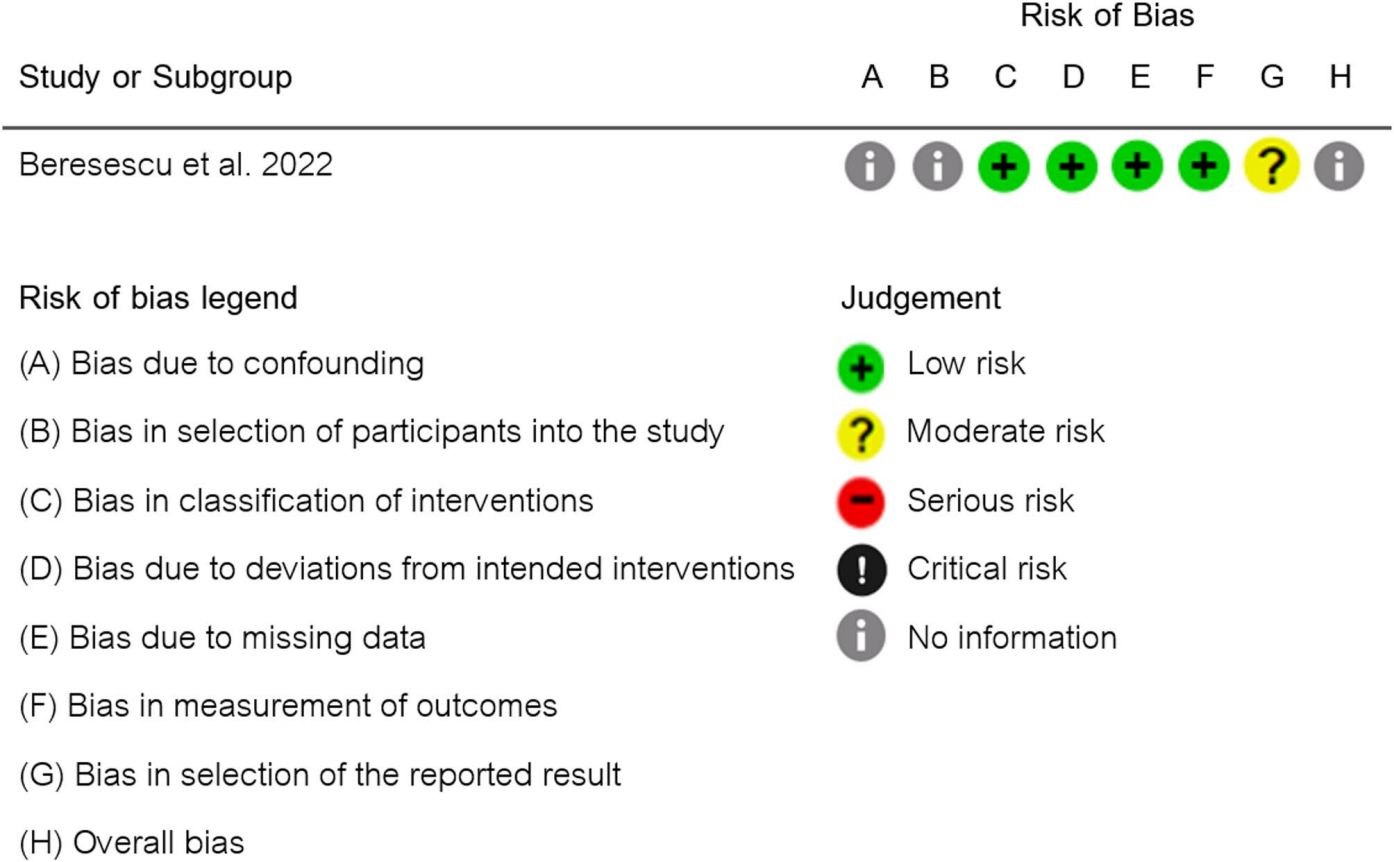
Risk of bias of non-randomized clinical trial

Most randomized clinical trials have shown a high risk of bias, primarily because deviations from the intended intervention could occur if control teeth (nonsealed) receive sealant treatment from external sources, and the use of unblinded evaluators could introduce measurement bias. Additionally, the lack of prespecified analysis plans raised concerns about reporting bias, preventing any trial from being categorized as low risk (Fig. 2). One nonrandomized trial provided no information in multiple domains due to its design, which lacked a comparative intervention (Fig. 3). For the cohort studies (Table 3), only one study was rated as good quality out of three. All risk of bias assessments are given in detail in Appendices B, C and D [see Additional file 1].

**Table 3:** Risk of bias of cohort studies^*a*^

Study	Selection				Comparability	Outcome			Total star (0–9)	Quality assessment
	S1	S2	S3	S4	C	O1	O2	O3		
Leskinen et al. 2008	*		*		*	*	*		5	Fair
Leskinen et al. 2008	*		*		*	*	*		5	Fair
Oulis & Berdouses 2009	*	*	*	*	*	*	*	*	9	Good

^a^The revised Cochrane risk-of-bias tool for randomized trials (RoB 2) for randomized clinical trials, the Risk of Bias in Nonrandomized Studies of Interventions (ROBINS-I) for clinical trials, and the Newcastle‒Ottawa Scale (NOS) for cohort studies

### Results of individual studies and data synthesis

#### Comparison among different caries risk groups (Table 1)

Based on caries incidence data, the results of three studies were transformed into relative risks (RR), indicating that high-caries-risk children (dmft > 4 or > 5) are at significantly higher risk of developing new carious lesions in sealed teeth. Specifically, Al-Jobair et al. [20] corresponds to an RR of 24.00 (95% CI: 3.39–169.92; *P* = 0.002) for high-caries-risk children compared with moderate-risk children at 18 months; Chen and Liu [21] corresponds to an RR of 8.89 (95% CI: 1.09–72.20; *P* = 0.041) for high-caries-risk children compared with low-risk children at 24 months; and Oulis and Berdouses [22] corresponds to an RR of 1.90 (95% CI: 1.34–2.69; *P* < 0.001) for high-caries-risk children compared with low-risk children at 36 months.

Muller-Bolla et al. [23, 24] analyzed both sealed and nonsealed teeth and reported that sealants significantly reduced the incidence of caries in children with active caries at baseline, with an odds ratio of 0.25 (95% CI: 0.12–0.50) at 12 months and a hazard ratio of 0.32 (95% CI: 0.23–0.46) at 36 months of follow-up. In contrast, sealants had negligible effects on those without active caries at baseline.

Leskinen et al. [25], using survival analysis, compared caries development outcomes at two health centers: Vantaa, where all children’s teeth were sealed regardless of caries risk, and Kemi, where only high-caries-risk children’s teeth were sealed. The results revealed that the incidence of caries was slightly lower in Kemi than in Vantaa. In a subsequent study [18] using the same population, Leskinen et al. assessed the cost-effectiveness of sealant strategies and found that the mean cumulative cost of sealant application and restorative treatment for all patients was lower in Kemi (184.20€ per subject) than in Vantaa (234.30€ per subject).

#### Caries development in the high-caries-risk group (Table 2)

Our analysis focused on caries development. Most studies assessed dentin caries via the ICDAS II code 4 or higher, the ART caries assessment codes 2–4, the WHO Oral Health Survey: Basic Methods (4th ed.), or unspecified assessment methods. For caries affecting both enamel and dentin, assessments included ICDAS II code 3 or higher, the color, coverage, and caries (CCC) system, a DIAGNOdent pen score of 20 or higher, or an undefined system.

Multiple studies have documented outcomes at the 12-month follow-up interval. Three studies focused on dentin caries, reporting caries incidence rates of 4.18% and 31.11% [26, 27] and a cumulative survival rate of 98.1% [28]. Additionally, four studies examined caries affecting both enamel and dentin, with incidences ranging from 0 to 9.48% [29–32].

For follow-up periods between 12 and 24 months, studies reported varying outcomes. With respect to dentin caries, one study reported a caries incidence of 6.88% at 18 months and 11.06% at 24 months [26]. Two additional studies at the 24-month period reported cumulative survival rates of 95.4% and 98.9% [28, 33]. For caries affecting both enamel and dentin, incidence rates ranged from 0 to 12.07% [29, 32, 34]. One study utilized a different metric, reporting a hazard ratio of 0.17 [35].

For long-term follow-up periods beyond 24 months, the incidence of dentin caries incidence ranged from 1.1 to 25% [36, 37]. Cumulative survival rates for dentin caries were reported to be 91.4% [28] and 96.4% [38] at different time points. For caries affecting both enamel and dentin, one study reported no caries at 36 months, and a 21.4% incidence at 48 months [32]. These findings demonstrate the variability in caries progression and assessment methods across extended follow-up periods.

## Discussion

This systematic review examined the preventive effects of resin-based dental sealants in children with high caries risk compared with those with moderate or low caries risk. Sealed teeth in high-caries-risk children had a greater risk of new carious lesions than did those in children with a lower risk. Nevertheless, sealants are still recommended for high-risk groups because of their significant effect on reducing the incidence of caries, whereas in low-risk groups, the preventive effect is insignificant.

All studies grouped or recruited high-caries-risk participants on the basis of caries experience, either alone via the dmft index [39, 40], or the presence of teeth with an ICDAS II code of 3 or higher at baseline [24], or in combination with other factors like caries risk assessment tools, including the AAPD [41], CAMBRA [42], or ADA [43]. Several studies found significant in the incidence of caries on sealed teeth between high- and low-caries-risk children, even when only caries experience is used for classification [20–22]. This finding aligns with that of Mejare et al. [44] who indicated that baseline caries prevalence is one of the most accurate predictors of future caries development. However, assessing these clinical factors requires dental professionals making it difficult to apply in community settings with limited dental workforces [45–47].

Our findings consistently supported sealant utilization in high-risk groups, in line with the results of past reviews [9–11]. The specific inclusion criteria of this systematic review, which focused on studies utilizing individual caries risk assessment, underscore the critical importance of dentin caries experience as a key factor in defining high-caries-risk individuals for dental sealant programs and general clinical practices. The variability among the included studies provided comprehensive information about the preventive effect of sealants in high-caries-risk groups.

However, the diverse follow-up periods and outcome measurements limit the comparability of results across studies, making it difficult to synthesize the data or conduct a meta-analysis. The limited number of low-bias studies was another limitation in obtaining concrete results. Studies with well-controlled interventions, blinding, and prespecified analysis plans are essential to strengthen the evidence base in this area.

Despite our efforts to include various study designs to gather more information on sealant effectiveness in high-risk groups within middle- and low-income countries, most studies comparing sealant effectiveness among different caries risk groups have been conducted in high-income countries [18, 20, 22–25], with only one study from China [21], an upper-middle income country [48]. Consequently, this may affect the external validity of the results, and the applicability of the findings to low- and middle-income countries remains unclear. Moreover, while caries experience alone has proven effective in classifying different caries risk groups, defining an appropriate threshold for high-risk classification remains challenging.

Future research should improve generalizability by including diverse populations, particularly those from low- and middle-income countries. A more standardized approach to risk classification and sealant effectiveness evaluation is essential to ensure consistency across studies, which will support more informed sealant placement decisions.

## Conclusion

Resin-based sealants are still recommended for high-caries-risk children. However, generalizability and an appropriate threshold for risk assessment remain unclear due to the limited number of studies from low- to middle-income countries and variability in risk assessment methods.

## Electronic supplementary material

Below is the link to the electronic supplementary material.

Supplementary Material 1

Supplementary Material 2

## Data Availability

The datasets used and/or analysed during the current study are available from the corresponding author on reasonable request.
