# Supplementary material for: Resin-based sealant effectiveness in high-caries risk children: a systematic review
Source: BMC Oral Health. 2025 May 23;25:768. doi: 10.1186/s12903-025-06158-0 (PMC12100864; doi:10.1186/s12903-025-06158-0)
Supplement: Supplementary file 1 — Supplementary Material 1 [file 12903_2025_6158_MOESM1_ESM.docx]

**Appendices**

**Appendix A. Search strategy for each database**

Pubmed

#1 "first grader*" or "primary school*" or "primary school student*" or "child*" or "student*"

#2 ("fissure" and "sealant*") or "dental sealant*"

#3 (caries prevalence) or (caries incidence) or (caries progression) or (tooth decay) or "caries occurrence*" or "dental caries" or "DMFT" or "ICDAS" or "cavit*"

#4 #1 and #2 and #3

Filters Clinical Study, Clinical Trial, Comparative Study, English, Thai, from 2000 - 2023

Cochrane Library

#1 (first grader*) or (primary school*) or (primary school student*) or (child*) or (student*) with Publication Year from 2000 to 2023, in Trials (Word variations have been searched)

#2 (fissure AND sealant*) or (dental sealant*) with Publication Year from 2000 to 2023, in Trials (Word variations have been searched)

#3 (caries prevalence) or (caries incidence) or (caries progression) or (tooth decay) or (caries occurrence*) or (dental caries) or (DMFT) or (ICDAS) or (cavit*) with Publication Year from 2000 to 2023, in Trials (Word variations have been searched)

#4 #1 AND #2 AND #3

Ovid Embase

1 ("first grader*" or "primary school*" or "primary school student*" or "child*" or "student*").mp. [mp=ti, ab, tx, kw, ct, ot, fx, sh, hw, bt, nm, kf, ox, px, rx, ui, sy, ux, mx]

2 (("fissure" and "sealant*") or "dental sealant*").mp. [mp=ti, ab, tx, kw, ct, ot, fx, sh, hw, bt, nm, kf, ox, px, rx, ui, sy, ux, mx]

3 (("caries" adj5 "prevalence") or ("caries" adj5 "incidence") or ("caries" adj5 "progression") or ("tooth" adj5 "decay") or "caries occurrence*" or "dental caries" or "DMFT" or "ICDAS" or "cavit*").mp. [mp=ti, ab, tx, kw, ct, ot, fx, sh, hw, bt, nm, kf, ox, px, rx, ui, sy, ux, mx]

4 1 and 2 and 3

5 limit 4 to (english or thai)

6 limit 5 to (clinical trial,all or clinical study or comparative study)

7 limit 6 to yr="2000 -Current"

8 limit 7 to humans

Google Scholar

1 Find articles with all of the words: child* and ("fissure sealant*" or "dental sealant*") and
("caries" or "decay*") and clinical study

2 Return articles dated between: 2000 - 2023

**Appendix B. Risk of bias judgement detail from each study (randomized clinical trial)**

**Table B.1** Al-Jobair et al. 2017

| **Bias** | **Authors’ judgement** | **Support for judgement** |
| --- | --- | --- |
| Bias arising from the randomization process | Low risk | Despite the lack of mentioned allocation concealment, the use of split-mouth designs with specific inclusion criteria and randomized allocation could minimize the bias. |
| Bias due to deviations from the intended interventions | Low risk | No deviations from the intended intervention arose due to the trial context. Modified intention-to-treat (mITT) analyses were employed, excluding participants with missing outcome data. |
| Bias due to missing outcome data | Low risk | The study had a 16.7% missing data rate, which was not dependent on the true values. |
| Bias in measurement of the outcome | Low risk | Measurements were appropriately conducted by blinded evaluators. |
| Bias in selection of the reported result | Some concerns | No information provided. |
| Overall bias | Some concerns | One domain in the results was identified as some concerns. |

**Table B.2** Amaechi et al. 2019

| **Bias** | **Authors’ judgement** | **Support for judgement** |
| --- | --- | --- |
| Bias arising from the randomization process | Low risk | Despite the lack of mentioned allocation concealment, the use of split-mouth designs with specific inclusion criteria and randomized allocation could minimize the bias. |
| Bias due to deviations from the intended interventions | Low risk | No deviations from the intended intervention arose due to the trial context. Modified intention-to-treat (mITT) analyses were employed, excluding participants with missing outcome data. |
| Bias due to missing outcome data | Low risk | The study had a 5% missing data rate, which was not dependent on the true values. |
| Bias in measurement of the outcome | High risk | Measurements were conducted without blinded evaluators. |
| Bias in selection of the reported result | Some concerns | No information provided. |
| Overall bias | High risk | One domain in the results was identified as high risk of bias. |

**Table B.3** Barja-Fidalgo et al. 2009

| **Bias** | **Authors’ judgement** | **Support for judgement** |
| --- | --- | --- |
| Bias arising from the randomization process | Some concerns | No information about allocation concealment and baseline differences between intervention groups. |
| Bias due to deviations from the intended interventions | Low risk | No deviations from the intended intervention arose due to the trial context. Modified intention-to-treat (mITT) analyses were employed, excluding participants with missing outcome data. |
| Bias due to missing outcome data | Low risk | The study had a 56% missing data rate, which was not dependent on the true values. |
| Bias in measurement of the outcome | Low risk | Measurements were appropriately conducted by blinded evaluators. |
| Bias in selection of the reported result | Some concerns | No information provided. |
| Overall bias | Some concerns | Two domains in the results were identified as some concerns. |

**Table B.4** Chen & Liu 2013

| **Bias** | **Authors’ judgement** | **Support for judgement** |
| --- | --- | --- |
| Bias arising from the randomization process | Low risk | Despite the lack of mentioned allocation concealment, the use of split-mouth designs with specific inclusion criteria and randomized allocation could minimize the bias. |
| Bias due to deviations from the intended interventions | Low risk | No deviations from the intended intervention arose due to the trial context. Modified intention-to-treat (mITT) analyses were employed, excluding participants with missing outcome data. |
| Bias due to missing outcome data | Low risk | The study had a 6.6% missing data rate, which was not dependent on the true values. |
| Bias in measurement of the outcome | High risk | Measurements were conducted without blinded evaluators. |
| Bias in selection of the reported result | Some concerns | No information provided. |
| Overall bias | High risk | One domain in the results was identified as high risk of bias. |

**Table B.5** Chen et al. 2012

| **Bias** | **Authors’ judgement** | **Support for judgement** |
| --- | --- | --- |
| Bias arising from the randomization process | Low risk | There were allocation concealment and randomized allocation without baseline differences between intervention groups. |
| Bias due to deviations from the intended interventions | Low risk | No deviations from the intended intervention arose due to the trial context. Modified intention-to-treat (mITT) analyses were employed, excluding participants with missing outcome data. |
| Bias due to missing outcome data | Low risk | The study had a 2.7% missing data rate, which was not dependent on the true values. |
| Bias in measurement of the outcome | Low risk | Measurements were appropriately conducted by blinded evaluators. |
| Bias in selection of the reported result | Some concerns | No information provided. |
| Overall bias | Some concerns | One domain in the results was identified as some concerns. |

**Table B.6** Gyati et al. 2023

| **Bias** | **Authors’ judgement** | **Support for judgement** |
| --- | --- | --- |
| Bias arising from the randomization process | Low risk | Despite the lack of mentioned allocation concealment, the use of split-mouth designs with specific inclusion criteria and randomized allocation could minimize the bias. |
| Bias due to deviations from the intended interventions | Low risk | No deviations from the intended intervention arose due to the trial context. Modified intention-to-treat (mITT) analyses were employed, excluding participants with missing outcome data. |
| Bias due to missing outcome data | Low risk | The study had no missing data. |
| Bias in measurement of the outcome | High risk | No information about evaluators provided. |
| Bias in selection of the reported result | Some concerns | No information provided. |
| Overall bias | High risk | One domain in the results was identified as high risk of bias. |

**Table B.7** Haricharan et al. 2019

| **Bias** | **Authors’ judgement** | **Support for judgement** |
| --- | --- | --- |
| Bias arising from the randomization process | Low risk | Despite the lack of mentioned allocation concealment, the use of split-mouth designs with specific inclusion criteria and randomized allocation could minimize the bias. |
| Bias due to deviations from the intended interventions | Low risk | No deviations from the intended intervention arose due to the trial context. Modified intention-to-treat (mITT) analyses were employed, excluding participants with missing outcome data. |
| Bias due to missing outcome data | Low risk | The study had no missing data. |
| Bias in measurement of the outcome | Low risk | Measurements were appropriately conducted by blinded evaluators. |
| Bias in selection of the reported result | Some concerns | No information about pre-specified analysis plan provided. |
| Overall bias | Some concerns | One domain in the results was identified as some concerns. |

**Table B.8** Haznedaroğlu et al. 2016

| **Bias** | **Authors’ judgement** | **Support for judgement** |
| --- | --- | --- |
| Bias arising from the randomization process | Low risk | There were allocation concealment and randomized allocation without baseline differences between intervention groups. |
| Bias due to deviations from the intended interventions | Low risk | No deviations from the intended intervention arose due to the trial context. Modified intention-to-treat (mITT) analyses were employed, excluding participants with missing outcome data. |
| Bias due to missing outcome data | Low risk | The study had a 40% missing data rate, which was not dependent on the true values. |
| Bias in measurement of the outcome | Low risk | Although the evaluators were not blinded, the outcome assessment could not be influenced by knowledge of intervention received. |
| Bias in selection of the reported result | Some concerns | No information about pre-specified analysis plan provided. |
| Overall bias | Some concerns | One domain in the results was identified as some concerns. |

**Table B.9** Hilgert et al. 2017

| **Bias** | **Authors’ judgement** | **Support for judgement** |
| --- | --- | --- |
| Bias arising from the randomization process | Some concerns | No information about allocation concealment, but had randomized allocation without baseline differences between intervention groups. |
| Bias due to deviations from the intended interventions | Low risk | No deviations from the intended intervention arose due to the trial context. Modified intention-to-treat (mITT) analyses were employed, excluding participants with missing outcome data. |
| Bias due to missing outcome data | Low risk | The study had a 30.1% missing data rate, which was not dependent on the true values. |
| Bias in measurement of the outcome | High risk | Measurements were conducted without blinded evaluators. |
| Bias in selection of the reported result | Some concerns | No information provided. |
| Overall bias | High risk | One domain in the results was identified as high risk of bias. |

**Table B.10** Kamath et al. 2022

| **Bias** | **Authors’ judgement** | **Support for judgement** |
| --- | --- | --- |
| Bias arising from the randomization process | Low risk | Despite the lack of mentioned allocation concealment, the use of split-mouth designs with specific inclusion criteria and randomized allocation could minimize the bias. |
| Bias due to deviations from the intended interventions | Low risk | No deviations from the intended intervention arose due to the trial context. Modified intention-to-treat (mITT) analyses were employed, excluding participants with missing outcome data. |
| Bias due to missing outcome data | Low risk | The study had a 6.5% missing data rate, which was not dependent on the true values. |
| Bias in measurement of the outcome | Low risk | Measurements were appropriately conducted by blinded evaluators. |
| Bias in selection of the reported result | Some concerns | No information provided. |
| Overall bias | Some concerns | One domain in the results was identified as some concerns. |

**Table B.11** Kervanto-Seppälä et al. 2008

| **Bias** | **Authors’ judgement** | **Support for judgement** |
| --- | --- | --- |
| Bias arising from the randomization process | Some concerns | The allocation sequence was chosen according to the child’s birth date, which is predictable. |
| Bias due to deviations from the intended interventions | Low risk | No deviations from the intended intervention arose due to the trial context. Modified intention-to-treat (mITT) analyses were employed, excluding participants with missing outcome data. |
| Bias due to missing outcome data | Low risk | The study had a 20% missing data rate, which was not dependent on the true values. |
| Bias in measurement of the outcome | High risk | No information about evaluators provided. |
| Bias in selection of the reported result | Some concerns | No information provided. |
| Overall bias | High risk | One domain in the results was identified as high risk of bias. |

**Table B.12** Muller-Bolla et al. 2013

| **Bias** | **Authors’ judgement** | **Support for judgement** |
| --- | --- | --- |
| Bias arising from the randomization process | Low risk | Despite the lack of mentioned allocation concealment, the use of split-mouth designs with specific inclusion criteria and randomized allocation could minimize the bias. |
| Bias due to deviations from the intended interventions | High risk | Deviations from the intended intervention may occur if control teeth receive sealant treatment from external sources. |
| Bias due to missing outcome data | Low risk | The study had an 8.3% missing data rate, which was not dependent on the true values. |
| Bias in measurement of the outcome | High risk | Measurements were conducted without blinded evaluators. |
| Bias in selection of the reported result | Some concerns | No information provided. |
| Overall bias | High risk | Two domains in the results were identified as high risk of bias. |

**Table B.13** Muller-Bolla et al. 2016

| **Bias** | **Authors’ judgement** | **Support for judgement** |
| --- | --- | --- |
| Bias arising from the randomization process | Low risk | Despite the lack of mentioned allocation concealment, the use of split-mouth designs with specific inclusion criteria and randomized allocation could minimize the bias. |
| Bias due to deviations from the intended interventions | High risk | Deviations from the intended intervention may occur if control teeth receive sealant treatment from external sources. |
| Bias due to missing outcome data | Low risk | The study had an 17.4% missing data rate, which was not dependent on the true values. |
| Bias in measurement of the outcome | High risk | Measurements were conducted without blinded evaluators. |
| Bias in selection of the reported result | Some concerns | No information provided. |
| Overall bias | High risk | Two domains in the results were identified as high risk of bias. |

**Table B.14** Muller-Bolla et al. 2018

| **Bias** | **Authors’ judgement** | **Support for judgement** |
| --- | --- | --- |
| Bias arising from the randomization process | Low risk | Despite the lack of mentioned allocation concealment, the use of split-mouth designs with specific inclusion criteria and randomized allocation could minimize the bias. |
| Bias due to deviations from the intended interventions | High risk | Deviations from the intended intervention may occur if control teeth receive sealant treatment from external sources. |
| Bias due to missing outcome data | Low risk | The study had a 27.5% missing data rate, which was not dependent on the true values. |
| Bias in measurement of the outcome | Low risk | Measurements were appropriately conducted by blinded evaluators. |
| Bias in selection of the reported result | Some concerns | No information provided. |
| Overall bias | High risk | One domain in the results was identified as high risk of bias. |

**Table B.15** Tahani et al. 2021

| **Bias** | **Authors’ judgement** | **Support for judgement** |
| --- | --- | --- |
| Bias arising from the randomization process | Low risk | There were allocation concealment and randomized allocation without baseline differences between intervention groups. |
| Bias due to deviations from the intended interventions | High risk | Deviations from the intended intervention may occur if control teeth receive sealant treatment from external sources. |
| Bias due to missing outcome data | Low risk | The study had a 12.1% missing data rate, which was not dependent on the true values. |
| Bias in measurement of the outcome | Low risk | Measurements were appropriately conducted by blinded evaluators. |
| Bias in selection of the reported result | Some concerns | No information about pre-specified analysis plan provided. |
| Overall bias | High risk | One domain in the results was identified as high risk of bias. |

**Table B.16** Zhang et al. 2014

| **Bias** | **Authors’ judgement** | **Support for judgement** |
| --- | --- | --- |
| Bias arising from the randomization process | Low risk | There were allocation concealment and randomized allocation without baseline differences between intervention groups. |
| Bias due to deviations from the intended interventions | Low risk | No deviations from the intended intervention arose due to the trial context. Modified intention-to-treat (mITT) analyses were employed, excluding participants with missing outcome data. |
| Bias due to missing outcome data | Low risk | The study had a 9.9% missing data rate, which was not dependent on the true values. |
| Bias in measurement of the outcome | Low risk | Measurements were appropriately conducted by blinded evaluators. |
| Bias in selection of the reported result | Some concerns | No information provided. |
| Overall bias | Some concerns | One domain in the results was identified as some concerns. |

Appendix C. Risk of bias judgement detail from each study (non-randomized clinical trial)

**Table C.1** Beresescu et al. 2022

| **Bias** | **Authors’ judgement** | **Support for judgement** |
| --- | --- | --- |
| Bias due to confounding | No information | Comparison of confounding between group was incapable due to only one intervention group. |
| Bias in selection of participants into the study | No information | No information whether start of follow-up and start of intervention coincide for most participants. |
| Bias in classification of interventions | Low risk | Clearly define sealant intervention on different baseline tooth status. |
| Bias due to deviations from intended interventions | Low risk | No deviations from the intended intervention beyond what would be expected in usual practice. |
| Bias due to missing data | Low risk | Outcome data were available nearly all participants, 95.8%. |
| Bias in measurement of outcomes | Low risk | The outcome measure was unlikely to be influenced by knowledge of the intervention received. |
| Bias in selection of the reported result | Moderate risk | There was no evidence of a pre-registered protocol or  statistical analysis plan. |
| Overall bias | No information | No clear indication that the study is at serious or critical risk of bias and there is a lack of information in one or more key domains of bias. |

Appendix D. Risk of bias judgement detail from each study (cohort study)

**Table D.1** Leskinen et al. 2008

| **Domain** | **Authors’ judgement** | **Support for judgement** |
| --- | --- | --- |
| Representativeness of the intervention cohort | 1 star | The subjects represented the whole age cohort born in 1988–1990. |
| Selection of the non intervention cohort | 0 star | Different sealant strategies were used in different towns. |
| Ascertainment of intervention | 1 star | The study was based on digital dental records. |
| Demonstration that outcome of interest was not present at start of study | 0 star | No information provided. |
| Comparability of cohorts on the basis of the design or analysis | 1 star | The study controlled for the participants' age and conducted separate analyses based on sex. |
| Assessment of outcome | 1 star | Outcome were assessed through database records. |
| Was follow up long enough for outcomes to occur | 1 star | 7 years follow-up with almost 70% of the age cohort was examined once a year. |
| Adequacy of follow up of cohorts | 0 star | The number of subjects lost to follow up was less than 20%. |
| Overall quality | Fair | 2 stars in selection domain and 1 star in comparability domain and 2 stars in outcome/exposure domain. |

**Table D.2** Leskinen et al. 2008 (CEA)

| **Domain** | **Authors’ judgement** | **Support for judgement** |
| --- | --- | --- |
| Representativeness of the intervention cohort | 1 star | The subjects represented the whole age cohort born in 1988–1990. |
| Selection of the non intervention cohort | 0 star | Different sealant strategies were used in different towns. |
| Ascertainment of intervention | 1 star | The study was based on digital dental records. |
| Demonstration that outcome of interest was not present at start of study | 0 star | No information provided. |
| Comparability of cohorts on the basis of the design or analysis | 1 star | The study controlled for the participants' age. |
| Assessment of outcome | 1 star | Outcome were assessed through database records. |
| Was follow up long enough for outcomes to occur | 1 star | 5 years follow-up with almost 70% of the age cohort was examined once a year. |
| Adequacy of follow up of cohorts | 0 star | The number of subjects lost to follow up was less than 20%. |
| Overall quality | Fair | 2 stars in selection domain and 1 star in comparability domain and 2 stars in outcome/exposure domain. |

**Table D.3** Oulis & Berdouses 2009

| **Domain** | **Authors’ judgement** | **Support for judgement** |
| --- | --- | --- |
| Representativeness of the intervention cohort | 1 star | Patients participating in the study were healthy children from all socio-economic levels. |
| Selection of the non intervention cohort | 1 star | Participant in every group were drawn from the same community. |
| Ascertainment of intervention | 1 star | The study was based on longitudinal data collected during the course of dental care in a private practice. |
| Demonstration that outcome of interest was not present at start of study | 1 star | Sealants were applied on sound tooth surfaces with no visible defects and/or on questionable surfaces. |
| Comparability of cohorts on the basis of the design or analysis | 1 star | Analysis models were adjusted with multiple variables. |
| Assessment of outcome | 1 star | Evaluators did not know who applied the sealant initially. |
| Was follow up long enough for outcomes to occur | 1 star | All the teeth were monitored for at least 3 years with at least one recall visit per year. |
| Adequacy of follow up of cohorts | 1 star | The number of subjects lost to follow up was less than 20%. |
| Overall quality | Good | 4 stars in selection domain and 1 star in comparability domain and 3 stars in outcome/exposure domain. |
