# Supplementary material for: Resin-based sealant effectiveness in high-caries risk children: a systematic review
Source: BMC Oral Health. 2025 May 23;25:768. doi: 10.1186/s12903-025-06158-0 (PMC12100864; doi:10.1186/s12903-025-06158-0)
Supplement: Supplementary file 2 — Supplementary Material 2 [file 12903_2025_6158_MOESM2_ESM.docx]

**Table 1** Studies with statistical analysis across different caries risk groups.

| **Authors, Year of publication** | **Study design** | **Study sample** | **Caries risk grouping criteria** | **Intervention (sealant brand)** | **Length of follow-up (months)** | **Outcome** |
| --- | --- | --- | --- | --- | --- | --- |
| **Al-Jobair et al., 2017 [20]** | Split-mouth randomized clinical trial | 42 children aged 6-9 years in Saudi Arabia (168 permanent first molars: free of caries, restorations, or sealant); 16.7% attrition | Moderate risk (dmft=1-4),  High risk (dmft>4) [22] | Moderate vs high caries risk in resin-based sealant (Clinpro™) and GI sealant (GC Fuji Triage) | 18 | Caries incidence (Oral health survey: basic methods 4^th^ed.) (Ref: resin sealant in moderate risk)  RR in high risk = 24.00 (3.39-169.92) ^a^ |
| **Chen & Liu, 2013 [21]** | Split-mouth randomized clinical trial | 61 children aged 6-8 years in China (158 permanent first molars: no caries found by visual inspection and probing); 6.6% attrition | Low risk (dmft<2),  High risk (dmft>5) [39, 40] | Low vs high caries risk in resin-based sealant (Concise) and GI sealant (GC Fuji VII) | 24 | Caries incidence (WHO) (Ref: resin sealant in low risk)  RR in high risk = 8.89 (1.09-72.20) ^a^ |
| **Muller-Bolla et al., 2016 [24];**  **Muller-Bolla et al., 2013 [23]** | Split-mouth randomized clinical trial | 276 children aged 6-7 years in France (914 permanent first molars: ICDAS II codes 0-2 without sealants); attrition 17.4% | Criteria followed Haute Autorite´ de Sante´ method [49] | Sealed teeth (Delton plus) vs non-sealed teeth | 36 | Caries incidence (ICDAS II codes 3-6) (Ref: non-sealed teeth)  HR in sealed teeth = 0.33 (0.24–0.46)  HR in sealed teeth of children with caries at baseline = 0.32 (0.23–0.46)  HR in sealed teeth of children without caries at baseline = 0.42 (0.16–1.12) |
|  | Split-mouth randomized clinical trial | 276 children aged 6-7 years in France (914 permanent first molars: ICDAS II codes 0-2 without sealants); attrition 8.3% | Criteria followed Haute Autorite´ de Sante´ method [49] | Sealed teeth (Delton plus) vs non-sealed teeth | 12 | Caries incidence (ICDAS II code 3-6) (Ref: non-sealed teeth)  OR in sealed teeth = 0.55 (0.37–0.81)  OR in sealed teeth of children with caries at baseline = 0.25 (0.12–0.50)  OR in sealed teeth of children without caries at baseline = 0.32 (0.06–1.63) |
| **Oulis & Berdouses, 2009 [22]** | Cohort | 380 children aged 6-8 years in Greece (1,274 permanent first molars: no decalcification or cavitation); attrition 12.5% | Low risk (dmft=0),  Moderate risk (dmft=1-4),  High risk (dmft>4)  (N/A) | Low vs moderate vs high caries risk (Delton®) | 36 | Caries incidence (N/A) (Ref: low risk)  RR in moderate risk = 1.41 (0.82- 2.43) ^a^  RR in high risk = 1.90 (1.34 -2.69) ^a^ |
| **Leskinen et al., 2008 [25];**  **Leskinen et al., 2008 [18]** | Retrospective cohort | 3816 children aged 6-7 years in Finland; attrition was not reported | Had signs of dental caries and high Streptococcus mutans level in saliva at the age of 2 years. (N/A) | Sealed teeth (Delton plus) vs non-sealed teeth in high-caries risk sealing strategy and all patient sealing strategy | 84 | Survival rate at 13 years of age (caries had reached dentin)  High-caries risk sealing strategy  sealed teeth ≈ 75% ^a^  non-sealed teeth ≈ 80% ^a^  All patient sealing strategy  sealed teeth ≈ 75% ^a^  non-sealed teeth ≈ 50% ^a^ |
|  | Retrospective cohort | 3816 children aged 5-7 years in Finland; attrition was not reported | Had signs of dental caries and high *Streptococcus mutans* level in saliva at the age of 2 years. (N/A) | High-caries risk sealing strategy vs all patient sealing strategy (Delton) | 60 | Cost of total treatment per subject (€)  High-caries risk sealing strategy = 184.2  All patient sealing strategy = 234.3 |
| dmft: Decayed, Missing, and Filled primary teeth, ICDAS II: International Caries Detection and Assessment System, WHO: World Health Organization, GI: Glass Ionomer, Ref: Reference group, N/A: Reference of the criteria was not mentioned in the paper  ^a^ **=** calculated from the incidence provided in the result of papers | | | | | | |

**Table 2** Studies that include participants with a high caries risk as part of their inclusion criteria.

| **Authors, Year of publication** | **Study design** | **Study sample** | **Caries risk inclusion criteria (Ref)** | **Intervention (sealant brand)** | **Length of follow-up (months)** | **Outcome** |
| --- | --- | --- | --- | --- | --- | --- |
| **Gyati et al., 2023 [34]** | Split-mouth randomized clinical trial | Children aged 6-9 years in India  (80 permanent first molars: ICDAS II code 0 and without any history of preventive treatment from the last 6 months on the respective teeth); attrition 0% | High caries risk status from AAPD caries risk assessment tools [41] | Hydrophilic sealant (Embrace WetBond) vs Hydrophobic sealant (Clinpro) | 18 | Caries progression (ICDAS II)  ICDAS II code 0 = 58 (72.5%)  ICDAS II code 1 = 18 (22.5%)  ICDAS II code 2 = 4 (5%)  Total caries incidence if specify at ICDAS II codes 3-6 = 0% ^a^ |
| **Beresescu et al., 2022 [26]** | Clinical trial | 119 children aged 6-8 years in Romania (427 permanent first molars: ICDAS II codes 0-3 without sealants or restorations); attrition 4.7% | High caries risk status from CAMBRA system [42] | Baselined ICDAS II (Helioseal F™) | 24 | Caries incidence (ICDAS II codes 4-6) from baselined ICDAS II codes 0-3  at 24 months = 45 (11.06%) ^a^  at 18 months = 28 (6.88%) ^a^  at 12 months = 17 (4.18%) ^a^ |
| **Kamath et al., 2022 [29]** | Split-mouth randomized clinical trial | 62 children aged 6-9 years in India (116 permanent first molars: no discoloration, restorations, cavitations or developmental defects); attrition 6.5% | dft = 3–6 (N/A) | Conventional (Delton FS+) vs nanofilled (Filtek Z350 flowable  nanocomposite) | 18 | Caries incidence (CCC)  Conventional and nanofilled resin sealant at 18 months = 14 (12.07%) ^a^  Conventional and nanofilled resin sealant at 12 months = 11 (9.48%) ^a^ |
| **Tahani et al., 2021 [30]** | Split-mouth randomized clinical trial | 124 children aged 7-9 years in Iran (370 permanent first molars: ICDAS II codes 0-2 without dental fluorosis, sealants or restorations); attrition 12.1% | primary molar dmft ≥ 1, not having dental visit at least once a year and not brushing at least once or twice every day [50] | Sealed (Prime-Dent) vs non-sealed | 12 | Caries incidence (ICDAS II codes 3-6) from baselined ICDAS II codes 0-2 Sealed teeth = 9 (5.52%) ^a^  Non-sealed teeth = 26 (15.95%) ^a^ |
| **Amaechi et al., 2019 [31]** | Split-mouth randomized clinical trial | 120 children aged 7-20 years in the United States (288 permanent molars or premolars: sound, or incipient caries limited to the enamel without sealants or restorations in pits and fissures); attrition 5% | Moderate to high caries risk status from ADA caries risk assessment tools [43] | Selenium-containing (DenteShield™) vs selenium-free (UltraSeal™ XT Plus) | 12 | Caries incidence (incipient/ cavitated caries)  Selenium-containing and selenium-free sealant = 0 (0%) |
| **Haricharan et al., 2019 [27]** | Split-mouth randomized clinical trial | 90 children aged 7-11 years in India (180 permanent mandibular first molars: no cavitated carious dentinal lesions or developmental anomalies); attrition 0% | DMFT ≥ 2, poor oral hygiene status, history of irregular dental visits and a high frequency of exposure to sugars (N/A) | Moisture-tolerant resin sealant (Embrace WetBond) vs ART sealant (Ketac Molar Easy Mix) | 12 | Caries incidence (Oral health survey: basic methods 4^th^ed.) (Simonsen score 2 or 4)  Resin sealant = 28 (31.11%)^a^ |
| **Muller-Bolla et al., 2018 [35]** | Split-mouth randomized clinical trial | 400 children aged 5-15 years in France (1,326 permanent molars: ICDAS II codes 0-2 without sealants or restorations); attrition 27.5% | At least one ICDAS II codes 3–6 lesion at baseline [24] | Sealed (Delton plus®) vs non-sealed | 24 | Caries incidence (ICDAS II codes 3-6) (Ref: non-sealed teeth)  HR in sealed teeth = 0.17 (0.15–0.20) |
| **Hilgert et al., 2017 [28]** | Randomized clinical trial | 123 children aged 6-7 years in Brazil (238 occlusal surfaces of permanent first molars: ICDAS II code 1 with medium or deep fissures, ICDAS II code 2, or ICDAS II code 3); attrition 30.1% | ≥2 cavitated dentine caries in primary molars assessed according to the ICDAS II index (N/A) | Resin-based sealant (Fluoroshield) vs ART sealant (Ketac Molar Easymix) | 36 | Cumulative survival (ICDAS II codes 4-6)  Resin sealant at 36 months = 91.4%  Resin sealant at 24 months = 95.4%  Resin sealant at 12 months = 98.1% |
| **Haznedaroğlu et al., 2016 [32]** | Randomized clinical trial | 40 children aged 7-10 years in Turkey (160 permanent first molars: DIAGNOdent-pen score<13); attrition 40% | a mean dft index ≥2 (N/A) | Resin-based sealant (Ultraseal XT®) vs GI sealant (Fuji Triage®) | 48 | Caries incidence (DIAGNOdent-pen score>20)  Resin sealant at 48 months = 12 (21.4%)  Resin sealant at 36 months = 0 (0%)  Resin sealant at 24 months = 0 (0%)  Resin sealant at 12 months = 0 (0%) |
| **Zhang et al., 2014 [38];**  **Chen et al., 2012 [33]** | Randomized clinical trial | 405 children aged 7-9 years in China (1,304 permanent first molars: no dentin caries lesion in pits and fissures); attrition 9.9% | a mean dmfs ≥ 2 (N/A) | Resin-based (Clinpro®) vs GI (Ketac Molar Easymix®) vs GI plus added energy vs glass carbomer sealant (Glass Carbomer®) | 48 | Cumulative survival (ART caries assessment code2-4)  Resin sealant = 96.4% |
|  | Randomized clinical trial | 407 children aged 7-9 years in China (1,352 permanent first molars: no dentin caries lesion in pits and fissures); attrition 2.7% | dmft ≥ 2 (N/A) | Resin-based (Clinpro®) vs GI (Ketac Molar Easymix®) vs GI plus added energy vs glass carbomer sealant (Glass Carbomer®) | 24 | Cumulative survival (ART caries assessment code2-4)  Resin sealant = 98.9% |
| **Barja-Fidalgo et al., 2009 [37]** | Randomized clinical trial | 36 children aged 6-8 years in Brazil (92 permanent first molars: sound occlusal surface or noncavitated enamel lesion); attrition 44.4% | primary molar dmft ≥ 2 (N/A) | Resin-based (Delton) vs GI sealant (Fuji IX) | 60 | Caries incidence (a cavity that had clearly penetrated the dentin or seen on the bitewing X-ray)  Resin sealant = 7 (25%) |
| **Kervanto-Seppälä et al., 2008 [36]** | Split-mouth randomized clinical trial | 599 children aged 12-16 years in Finland (2,356 second permanent molars: no sealants, dentin caries, or restorations); attrition 20% | earlier dentin caries or increased caries activity of a sibling (N/A) | Resin-based (Delton®) vs GI sealant (Fuji III®) | 36 | Caries incidence (dentin caries  could be detected)  Resin sealant = 7 (1.1%) |
| dmft: Decayed, Missing, and Filled primary Teeth, dft: Decayed and Filled primary Teeth, dmfs: Decayed, Missing, and Filled Surfaces, DMFT: Decayed, Missing, and Filled permanent Teeth, ICDAS II: International Caries Detection and Assessment System, GI: Glass Ionomer, ART: Atraumatic Restorative Treatment, AAPD: American Academy of Pediatric Dentistry, ADA: American Dental Association, CAMBRA: Caries Management by Risk Assessment, Ref: Reference group, N/A: Reference of the criteria was not mentioned in the paper  ^a^ **=** calculated from the incidence provided in the result of papers | | | | | | |
